# The Role of Magnetic Resonance Imaging in Preoperative Evaluation of Anterior Obliterative Urethral Strictures

**DOI:** 10.3390/diagnostics15192415

**Published:** 2025-09-23

**Authors:** Kursat Kucuker, Duran Duzgun, Burak Saglam, Ilker Gokcedag, Mehmet Kirdar, Yusuf Ozlulerden, Sinan Celen, Mesut Berkan Duran, Ahmet Baki Yagci, Zafer Aybek

**Affiliations:** 1 Department of Urology, School of Medicine, Pamukkale University, 20160 Denizli, Turkey; 2 Department of Urology, Kütahya City Hospital, 43050 Kütahya, Turkey; 3 Department of Radiology, School of Medicine, Pamukkale University, 20160 Denizli, Turkey

**Keywords:** magnetic resonance imaging, anterior urethral stricture, preoperative assessment, urethroplasty

## Abstract

**Background/Objectives:** Conventional imaging modalities are often inadequate for evaluating the proximal extent of anterior obliterative urethral strictures. Magnetic Resonance Imaging (MRI), with its superior soft tissue resolution, provides detailed anatomical insights and significantly contributes to surgical planning in such cases. **Methods:** Four male patients aged 26–63 years with anterior obliterative urethral strictures were evaluated using MRI in addition to conventional imaging. All MRI scans were performed following a modified Joshi protocol. Clinical data, MRI findings, and surgical outcomes were retrospectively reviewed. **Results:** MRI successfully delineated stricture length, location, periurethral fibrosis, and proximal urethral status in all cases, correlating well with intraoperative findings. Case 1 showed a 2 cm proximal bulbar obliteration, excised with end-to-end anastomosis. Case 2 had a 2.5 cm distal bulbar stricture, managed similarly. Case 3 revealed multi-segmental strictures, treated with a combination of anastomosis, graft, and Kulkarni urethroplasty. Case 4 demonstrated a rare 9 cm distal penile obliteration with preserved proximal urethra, treated with anastomotic repair. MRI provided critical anatomical detail for surgical decision-making. **Conclusions:** MRI is a valuable imaging modality for the evaluation of anterior obliterative urethral strictures, particularly when the proximal extent of the stricture cannot be visualized with conventional imaging techniques. In our case series, MRI enabled precise delineation of the stricture length and surrounding anatomical structures, which was critical for selecting the most appropriate surgical approach.

## 1. Introduction

A urethral stricture is fundamentally a fibrotic process involving the urethral epithelium and the surrounding corpus spongiosum, which ultimately results in a reduction in or complete obliteration of the urethral lumen [1].

The male urethra is divided into anterior (bulbar, penile, navicular) and posterior (prostatic, membranous) segments. Strictures most commonly affect the anterior urethra, especially the bulbar part. The LSE classification (Length, Site, Etiology) is a useful framework to describe strictures and guide treatment [2]. Anterior obliterative urethral strictures represent a complex subset of urethral pathology and are typically caused by iatrogenic factors (e.g., catheterization, instrumentation), traumatic injuries, idiopathic mechanisms, or chronic inflammatory conditions such as lichen sclerosus [3,4,5]. Since complete obliterative anterior urethral strictures cannot be treated endoscopically, accurate preoperative assessment of the stricture length and its anatomical extent is essential for selecting the optimal open surgical approach and achieving successful outcomes [6].

The combination of A-RUG (antegrade–retrograde urethrography) is one of the most commonly used methods for preoperative evaluation of obliterative urethral strictures [7]. Current literature reports that retrograde urethrogram (RUG) provides 75–100% sensitivity and 72–97% specificity for diagnosis [8]. However, insufficient bladder neck opening, may limit the ability of these methods to accurately assess the length of strictures and its relationship to surrounding tissues [7]. In addition, RUG also has some limitations in the evaluation of spongiofibrosis [9]. Furthermore, proper interpretation of RUG requires extensive knowledge of normal urethral anatomy [10].

In recent years, advanced imaging techniques such as sonourethrography (SUG) and magnetic resonance urethrography (MRU) have gained increasing clinical relevance and are now recognized as valuable diagnostic tools in the assessment of urethral strictures. These modalities offer detailed information on the location and extent of the stricture, as well as the presence of spongiofibrosis and other periurethral pathologies [11].

Although urethral MRI is less commonly preferred, primarily due to its higher cost and limited availability in some settings, it provides critical insights into the evaluation of urethral tissues, periurethral soft tissues, spongiofibrosis, urethral cavity, stricture length and the presence of multiple urethral strictures. Due to its longer examination time and the requirement for specialized interpretation skills, MRI is used less frequently than other modalities such as RUG. MRI offers a clear view of the relationship between the urethra and adjacent structures, such as the symphysis pubis and rectum [4]. It was found that MRI measurements had a higher correlation with intraoperative measurements compared to combined anterograd-retrograd urethrography (A-RUG) [12]. With its high soft tissue resolution, MRI allows observation of urethral and periurethral tissues without radiation exposure [3].

Urethroplasty is considered the gold standard treatment for urethral strictures due to its high long-term success rates. However, in addition to surgical expertise, achieving optimal outcomes requires meticulous preoperative assessment and planning. Given the decreasing success rates of repeated interventions, the initial surgical approach is particularly critical. A well-planned first procedure can significantly reduce the risk of recurrence and complications. In this context, MRI plays a pivotal role by providing detailed visualization of the stricture, surrounding tissues, and any additional strictures, thereby enhancing the accuracy of the initial intervention and minimizing the need for subsequent procedures [13].

This case series aimed to show the added value of MRI in evaluating obliterative anterior urethral strictures before surgery. To illustrate this, we presented four selected cases, including their preoperative evaluations, surgical management, and clinical outcomes.

## 2. Materials and Methods

Informed consent was obtained from all four patients for the use of their patient information in this manuscript. Ethical approval was granted by the Pamukkale University Faculty of Medicine Ethics Committee (approval date: 24 December 2024, approval number: E-60116787-020-629689).

Patients included in this case series were those diagnosed with anterior obliterative urethral strictures between January 2024 and June 2025, who underwent both conventional imaging and urethral MRI as part of their preoperative assessment.

MRI scans were performed using a 1.5 Tesla superconducting magnet (Ingenia; Philips Healthcare, Best, The Netherlands) with a gradient strength of 45 mT/m on each axis and a maximum slew rate of 200 mT/m/s. A 32-channel anterior torso coil placed over the patient.

The protocol included 3D sagittal T2-weighted images and fat-suppressed sagittal 3D T1-weighted images acquired both before and after intravenous administration of 0.1 mmol/kg (0.2 mL/kg) gadoteric acid.

For urethral MRI, we employed the “Joshi protocol,” as described by Joshi et al., which has been shown to provide a more accurate depiction of the urethral gap compared to conventional urethrography [14]. On the night prior to imaging, patients were premedicated with 0.4 mg oral tamsulosin, a selective alpha-blocker, to facilitate adequate bladder neck relaxation. On the day of imaging, in cases with an existing cystostomy, the bladder was filled with sterile saline via the suprapubic catheter. Subsequently, a mixture of sterile saline and lignocaine jelly was instilled retrogradely through the urethral meatus, and the penis was gently clamped to maintain distension. This technique optimizes visualization of the entire urethral course and enhances anatomical delineation, particularly of the obliterated segment and the proximal urethra.

All MRI images were reviewed by an experienced radiologist and a urologist, with the urologist informed of the MRI findings prior to surgery. All operations were performed by the same reconstructive urologist. Postoperative outcomes were assessed using Qmax values from uroflowmetry at the 6th months.

## 3. Results

In the first case, MRI revealed a completely obliterative anterior urethral stricture with no additional proximal narrowing. Surgical findings were fully concordant with the preoperative imaging, and the patient was treated successfully with end-to-end anastomotic urethroplasty. Similarly, in the second case, MRI excluded the presence of any additional strictures proximal to the obliterated segment, and intraoperative findings confirmed this. The patient underwent an end-to-end anastomosis with favorable postoperative outcomes.

The third case involved a patient with a complex anterior urethral stricture, initially presumed to be traumatic in origin. Preoperative MRI demonstrated two completely obliterative strictures located at 4 cm and 7 cm from the meatus, along with a narrowed but patent proximal segment extending toward the sphincter. These findings were corroborated during surgery, which revealed three distinct stenotic regions: two completely obliterative strictures and a third, non-obliterative narrowing involving the bulbar urethra and proximal segment. A dorsolateral Kulkarni urethroplasty was performed for the bulbar segment, a double-face graft for the mid-segment, and an anastomotic urethroplasty for the distal stricture. The correlation between MRI findings and intraoperative observations highlights the role of MRI in identifying multiple-level strictures and tailoring complex surgical repairs accordingly.

In the fourth case, a patient with a history of pelvic trauma was initially presumed to have a posterior urethral stricture. However, MRI revealed a completely obliterative anterior stricture located 9 cm proximal to the meatus, while the proximal urethra remained unaffected. The patient was successfully treated with primary end-to-end anastomosis.

These four cases illustrate the utility of MRI in characterizing obliterative urethral strictures, particularly in determining the presence or absence of additional proximal pathology. The strong correlation between MRI and intraoperative findings across all cases underscores the modality’s value in preoperative assessment and surgical decision-making. A detailed summary of the patient characteristics, imaging findings, and surgical procedures is provided in Table 1.

Case 1:

A 62-year-old male patient, followed up with a catheter due to urethral obstruction following coronary bypass surgery in September 2023, In December 2023, he underwent internal urethrotomy due to urethral stricture. After recurring of stricture, a suprapubic catheter was inserted. Retrograde urethrography revealed that minimal passage of contrast from the posterior urethra to the bladder.

**Figure 1:**
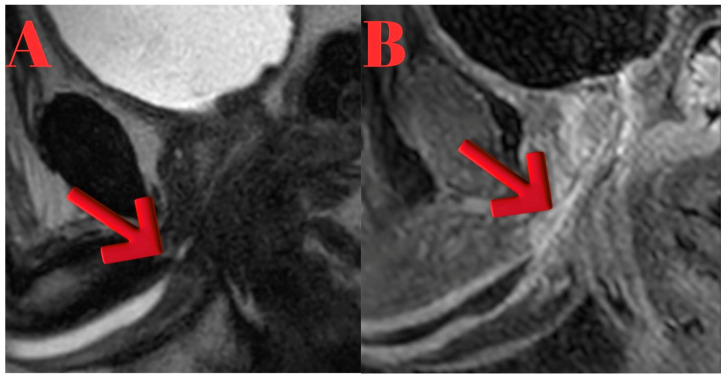
(**A**): Sagittal T2-weighted MRI showing inflammatory changes and complete obliteration extending from the proximal bulbar urethra to the distal prostatic urethra (red arrow indicating the obliterated segment). (**B**): Sagittal T1-weighted fat-suppressed post-contrast MRI highlighting fibrosis with contrast enhancement in the strictured segment (red arrow indicating the fibrotic lesion).

MRI findings showed lidocaine gel expanding the urethra up to the proximal bulbous urethra. T2 sagittal images demonstrated occlusion extending from the proximal bulbous urethra to the distal prostatic urethra. Post-contrast T1a sagittal imaging revealed a 1.5 cm complete obstruction in the distal prostatic urethra and a 5 mm partial stricture in the proximal bulbous urethra. Thickening of the urethral wall and enhanced inflammatory staining were noted. T1 sagittal imaging identified a 2 cm stenotic occlusive stricture area in the proximal bulbous urethra (Figure 1).

**Figure 2:**
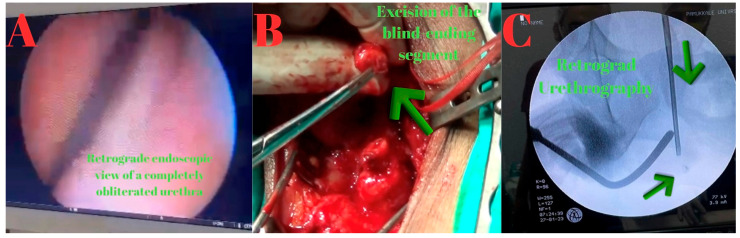
(**A**): Retrograde endoscopic view demonstrating a completely obliterated urethral lumen. (**B**): Intraoperative photograph obtained from surgical video, showing excision of the obliterated segment (green arrow). (**C**): Fluoroscopic view during combined assessment: antegrade access with a ureterorenoscope (green arrow) and retrograde insertion of a bougie dilator (green arrow), used simultaneously to delineate the gap between the proximal and distal urethral ends.

In March 2024, an anastomotic urethroplasty was performed. No antegrade or retrograde passage of guide or contrast was achieved. Through a perineal vertical incision, as demonstrated by the MRI, the fibrotic area consistent with a 2 cm complete stricture in the proximal bulbar urethra was excised, followed by end-to-end anastomosis (Figure 2).

Case 2:

In November 2022, a 63-year-old male patient, who had been followed up with a catheter after a liver transplant, underwent suprapubic cystostomy placement due to unsuccessful catheter insertion two weeks prior.

**Figure 3:**
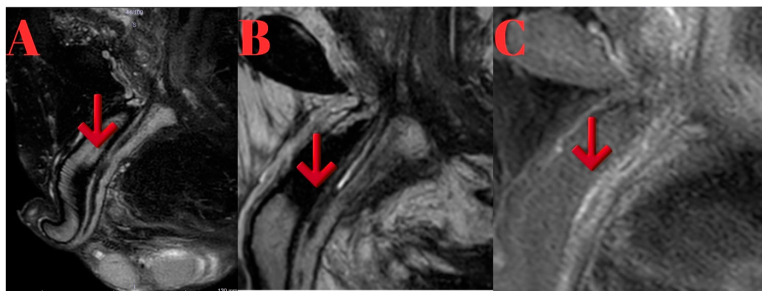
(**A**,**B**): T2-weighted sagittal images demonstrating complete obliteration at the distal bulbar urethra (arrows). (**C**): Post-contrast fat-suppressed T1-weighted sagittal image showing a narrowed lumen with surrounding inflammatory and fibrotic changes (arrow).

In MRI T1 and T2 imaging revealed two stricture areas in the distal bulbous urethra. Post-contrast, fat-suppressed T1a sagittal images demonstrated approximately a 2 cm lumen with moderate enhancement around the distal bulbous urethra, indicative of inflammation (Figure 3).

**Figure 4:**
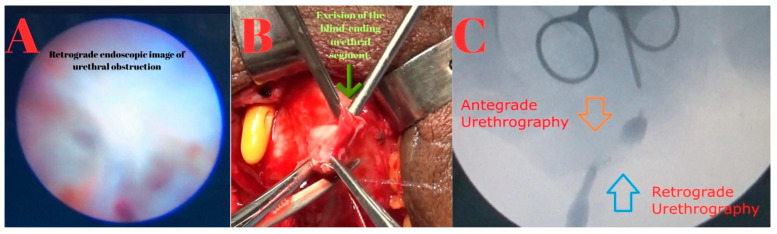
(**A**): Retrograde endoscopic image showing complete urethral obstruction. (**B**): Intraoperative photograph demonstrating excision of the blind-ending urethral segment (green arrow). (**C**): Combined antegrade (orange arrow) and retrograde (blue arrow) urethrography images, illustrating the non-communicating gap between the two ends of the urethra.

Retrograde urethrography and urethroscopy were performed first, followed by antegrade urethrography and urethroscopy through the suprapubic tract. Consistent with MRI findings, a 2.5 cm complete stricture was identified in the bulbous urethra. The stricture segment was excised, and an end-to-end anastomotic urethroplasty was performed (Figure 4).

Case 3:

A 58-year-old male patient presented to the urology department with recurrent urinary flow reduction following a work-related pelvic trauma 42 years prior (dynamite explosion). The patient was diagnosed with urethral stricture based on endoscopic evaluations, and multiple endoscopic interventions had been performed over the years. He had no known comorbidities and last underwent urethral dilatation three years prior. A suprapubic catheter (cystofix) was placed two months prior due to urinary retention.

Retrograde and antegrade urethrography revealed no passage of contrast between the penile urethra and the proximal segments. While antegrade contrast reached the prostatic urethra, the intervening segment could not be adequately visualized.

**Figure 5:**
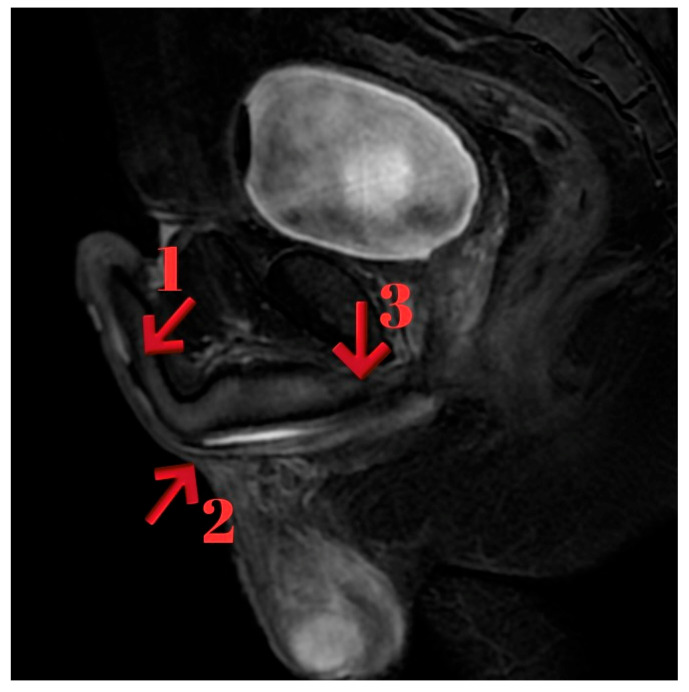
Arrow 1: Completely obliterated distal penile urethral stricture (4 cm from the meatus). Arrow 2: Second completely obliterated stricture located proximally at 7 cm from the meatus. Arrow 3: Additional non-obliterative narrowing within the bulbar urethra, extending toward the sphincter.

Urethral MRI, performed after bladder filling via the cystofix catheter, demonstrated that the lidocaine gel instilled through the meatus failed to advance through the penile urethra. Two focal fibrotic stenotic-occlusive lesions, each approximately 7 mm in length, were identified at 4 cm and 7 cm distal to the meatus in the distal penile urethra. A 6 mm localized dilatation was noted just proximal to these lesions in the penile urethra. The proximal penile urethra, bulbar urethra, and membranous urethra appeared patent. In the distal segment of the prostatic urethra, inadequate luminal expansion was observed over a 7 mm segment. However, the overall wall structures of the prostatic and membranous urethra were within normal limits. No posterior urethral obstruction was present (Figure 5).

**Figure 6:**
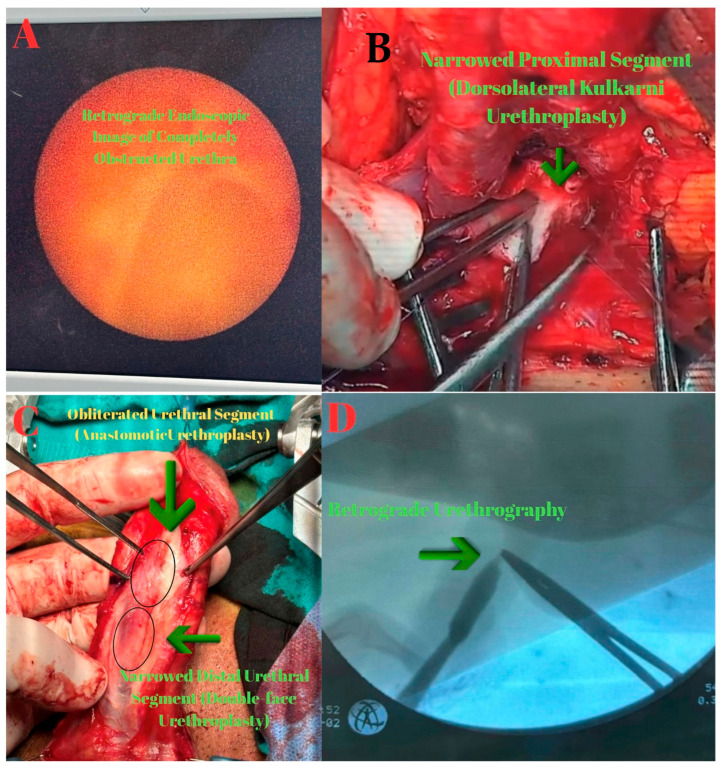
(**A**): Retrograde endoscopic image showing a completely obliterated urethral lumen. (**B**): Intraoperative view of the narrowed proximal segment, treated with dorsolateral Kulkarni urethroplasty (green arrow). (**C**): Intraoperative image showing the distal segment treated with anastomotic urethroplasty (green arrow) and, proximally, the adjacent mid-urethral area reconstructed with double-face urethroplasty (green arrow). (**D**): Retrograde urethrography highlighting the most distal obliterative lesion (green arrow).

Given the complexity and multi-segmental nature of the obliterative anterior stricture, a staged urethroplasty combining different surgical techniques was performed (Figure 6).

This comprehensive reconstructive approach was selected based on precise preoperative mapping provided by MRI.

Case 4:

In June 2024, a 26-year-old male patient with no known comorbidities was admitted to the emergency department following a motorcycle accident, with a suspected femur fracture. Due to the absence of urine output, retrograde urethrography was performed to rule out possible urethral or bladder trauma. Upon observing the disruption of urethral integrity, the patient and his relatives were informed about the delayed urethral repair. A cystostomy was performed, and the patient was subsequently followed up and treated by other relevant departments for associated multitrauma.

**Figure 7:**
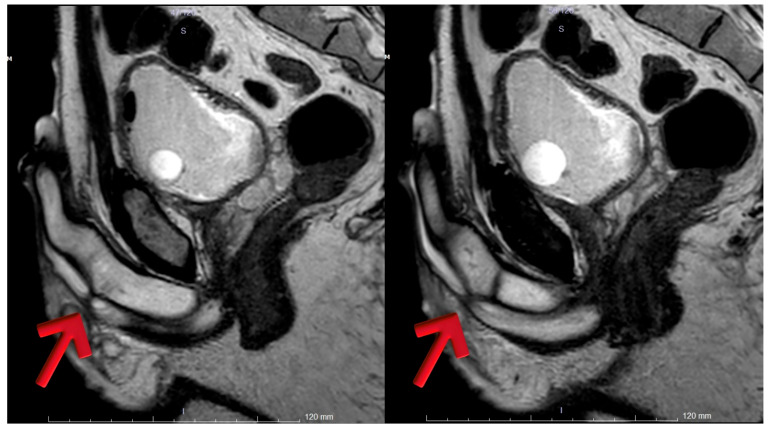
Sagittal T2-weighted MR images of Case 4. The completely obliterative anterior urethral stricture (arrow) is demonstrated at ~9 cm from the meatus. Two adjacent slices are presented to illustrate the precise location and extent of the stenotic segment, with preserved proximal urethra.

Five months after the accident, a urethral MRI revealed a focal stenotic-occlusive lesion in the penile urethra, approximately 9 cm distal to the urethral meatus. Proximal to the lesion, a localized dilation of up to 6 mm was observed in the penile urethra, with no other abnormalities noted in the remaining urethra (Figure 7).

**Figure 8:**
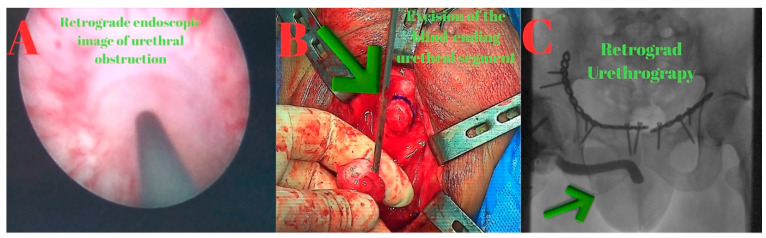
(**A**): Retrograde endoscopic image demonstrating complete urethral obstruction. (**B**): Intraoperative photograph showing excision of the blind-ending obliterated segment during urethroplasty (arrow). (**C**): Retrograde urethrography image, highlighting the obliterated segment in the anterior urethra (arrow).

Consequently, seven months after the accident, the patient was scheduled for anastomotic urethroplasty. During the procedure, retrograde urethrography showed no contrast passage to the posterior urethra. Endoscopy revealed a completely obliterated stricture starting approximately 9 cm from the urethral meatus. Simultaneously, a half-moon needle bougie was introduced through the existing cystostomy tract, and under fluoroscopy, the length of the strictured segment was determined to be less than 2 cm. A guidewire was then passed through the cystostomy tract, and a flexible cystoscope was advanced over the guidewire, revealing normal bladder, prostatic, and bulbar urethral anatomy. The fibrotic strictured segment was excised and removed. The freed proximal and distal ends of the urethra were spatulated, and anastomotic urethroplasty was performed (Figure 8).

At the 6-month postoperative follow-up, all four patients demonstrated marked improvement in urinary flow rates. Postoperative Qmax values were recorded as 27.6 mL/s in Case 1, 32.1 mL/s in Case 2, 23.4 mL/s in Case 3, and 29.2 mL/s in Case 4. At the 6-month follow-up, uroflowmetry curves were normal in all patients. These findings reflect successful anatomical reconstruction and satisfactory functional outcomes across a spectrum of complex anterior urethral strictures.

## 4. Histopathological Findings

In all four patients, excised urethral segments were subjected to histopathological evaluation. The predominant findings included dense collagen deposition and fibrosis consistent with obliterative strictures. In one patient, keratinizing squamous metaplasia and chronic active inflammation were also observed. These histological results were in line with the MRI findings, where areas of periurethral fibrosis and poor luminal expansion corresponded to fibrotic tissue confirmed on pathology. Thus, histopathological analysis supported the diagnostic accuracy of MRI in characterizing stricture-associated tissue changes.

## 5. Discussion

Urethral stricture is a pathological narrowing of the urethral lumen that can lead to significant lower urinary tract symptoms, urinary retention, and recurrent infections, severely impairing quality of life [15]. Diagnosis typically involves retrograde urethrography and combined A-RUG. Additional imaging modalities such as sonourethrography, CT and MRI provide valuable information in complex cases, particularly when the proximal extent of obliterative strictures cannot be clearly assessed or when multiple strictures are present [10].

In this case series, MRI proved valuable in preoperative planning by accurately showing stricture length, location, proximal urethral status, and any additional strictures. In all four cases, MRI findings matched surgical observations: two patients had isolated obliterations, one had multi-segmental strictures requiring combined techniques, and one had a rare post-traumatic penile obliteration with a preserved proximal urethra. These findings helped guide the surgical approach and prevent unexpected intraoperative challenges. Unlike conventional urethrography, MRI allowed for clear visualization of periurethral fibrosis, proximal patency, and stricture multiplicity.

MRI’s multiplanar capability and superior soft tissue resolution make it particularly advantageous in evaluating obliterative strictures and traumatic injuries [5,16]. Unlike sonourethrography, which is operator-dependent and may not visualize the entire urethra, or CT, which lacks soft tissue resolution, MRI offers a comprehensive anatomical overview [3,17]. Its utility has also been shown in female patients and those with periurethral cystic pathology, enhancing diagnostic and surgical accuracy [18].

In the interpretation of urethral MRI, the experience of the radiologist plays a crucial role. While an experienced radiologist can provide valuable insights, a report written by an inexperienced individual may lead to unexpected findings during the surgical procedure, potentially resulting in incorrect surgical planning. This underscores the importance of a multidisciplinary approach, involving both urologists and radiologists, to ensure the most accurate interpretation and optimal surgical planning [19,20].

One challenge in urethral MRI is the lack of standardized imaging protocols and parameters. While T2-weighted imaging is generally preferred, the specific sequences, image planes, and technical settings can vary significantly between institutions and radiologists. This variability may result in differences in diagnostic accuracy and limit the assessment of critical anatomical features. As with multiparametric MRI in prostate imaging—where standardization has significantly improved diagnostic consistency—the development and adoption of uniform urethral MRI protocols are essential to ensure reliable and reproducible results across clinical settings [21].

While retrograde urethrography remains the standard initial diagnostic tool, MRI is increasingly preferred for complex cases and preoperative planning and may potentially improve surgical outcomes [22]. These four cases findings reveal the decisive role of MRI in the diagnosis and treatment of urethral strictures.

In a study conducted by Sudhakar et al., magnetic resonance urethrography demonstrated 100% sensitivity and specificity in the evaluation of anterior urethral strictures. In contrast, RUG showed 80% sensitivity and only 33% specificity. These findings underscore the significantly greater diagnostic accuracy of MRI in identifying and characterizing strictures [23]. Consistent with our own findings, this study reinforces the superiority of MRI as an imaging modality for anterior urethral strictures. MRI offers more precise and comprehensive anatomical information, which directly impacts surgical decision-making and overall patient management.

Another method for evaluating urethral strictures is sonourethrography. McAninch et al. demonstrated that sonourethrography is capable of detecting underlying spongiofibrosis, which altered the estimated stricture length in 45% of patients when compared to urethrography [24]. A separate study involving 40 patients demonstrated that sonourethrography provided accurate assessments of anterior urethral strictures and yielded more detailed information than conventional urethrography [25]. Sonourethrography has also been found to be comparable to magnetic resonance urethrography, suggesting that MRI may not be necessary for evaluating the anterior urethra in most cases [26]. Although sonourethrography has shown comparable results to MRI in the evaluation of the anterior urethra, its operator dependency and limited utility in assessing the posterior urethra make MRI a more advantageous modality in patients where the proximal extent of the stricture cannot be adequately visualized, as demonstrated in our study.

Our findings are consistent with prior literature. For example, Mangera et al. reported that MRI findings altered surgical management in over 30% of anterior urethral stricture cases [27]. Similarly, Felix et al. demonstrated that MRI measurements of stricture length correlate more closely with intraoperative findings than retrograde urethrography, reinforcing MRI’s role in precise surgical planning [12,22]. In our study, all four cases showed high concordance between MRI and intraoperative findings, which allowed for tailored reconstruction strategies. Particularly in Case 3, the detection of multi-segmental involvement facilitated the decision to use three different reconstructive techniques.

These cases reveal that MRI is an effective tool for both detailed anatomical evaluation and surgical planning in the management of obliterative anterior urethral strictures. The use of such advanced imaging methods will increase the success rate of surgical results.

In each case, preoperative MRI findings were not only consistent with intraoperative observations but also guided the choice of surgical technique, ranging from anastomotic urethroplasty to staged repairs combining graft- and flap-based methods.

While MRI offers detailed anatomical visualization and clear advantages in the preoperative evaluation of anterior obliterative urethral strictures, its wider adoption is still limited by factors such as cost, availability, and the lack of standardized imaging protocols. Nevertheless, when available, MRI can serve as a powerful adjunct to conventional imaging, providing critical insights that may improve surgical planning and outcomes.

By enabling precise delineation of the stricture’s proximal extent and surrounding fibrosis, MRI allows surgeons to plan the most appropriate approach in advance, which can minimize operative time, reduce tissue trauma, and lower the risk of residual or recurrent strictures [28].

## 6. Limitations

This study underscores the potential role of MRI in the evaluation and surgical planning of anterior obliterative urethral strictures. However, several limitations must be acknowledged. First, the retrospective design and the small sample size limit the generalizability of the findings and increase the risk of selection bias. Additionally, the lack of direct comparisons with other imaging modalities, such as sonourethrography or computed tomography, precludes a comprehensive assessment of MRI’s relative advantages. Moreover, no quantitative statistical analysis was performed to correlate MRI findings with surgical outcomes, which weakens the ability to draw definitive conclusions. Finally, the limited duration of postoperative follow-up restricts the assessment of long-term efficacy and recurrence. These limitations emphasize the need for larger, prospective, and comparative studies with standardized imaging protocols and long-term follow-up to validate and expand upon these preliminary findings.

## 7. Conclusions

MRI proved to be a reliable and informative tool for the preoperative assessment of anterior obliterative urethral strictures, accurately defining stricture length, location, and complexity. The strong concordance between MRI and intraoperative findings underscores its value in guiding surgical planning, particularly when conventional imaging is insufficient. By delineating anatomical challenges preoperatively—including proximal patency and additional strictures—MRI enables tailored reconstruction strategies, reduces operative complexity and duration, and ultimately improves clinical outcomes. Therefore, MRI should be considered a key adjunct in the preoperative evaluation of selected anterior obliterative urethral strictures.

## Figures and Tables

**Table 1:** Summary of Clinical, Radiological, and Surgical Characteristics of the Patients.

Case	Age	Gender	Disease History	MRI Findings	Process	Surgical Intervention and Results	LSE Classification
1	62	Male	Coronary bypass (Sep 2023), urethral obstruction, internal urethrotomy (Dec 2023), recurrent stricture, suprapubic catheter placement	Lidocaine gel expanded urethra up to proximal bulbous urethra. T2 sagittal: Occlusion from proximal bulbous urethra to distal prostatic urethra. Post-contrast T1a sagittal: 1.5 cm complete occlusion (distal prostatic urethra) + 5 mm partial stricture (proximal bulbous urethra). T1 sagittal: 2 cm obstructive stricture in proximal bulbous urethra.	Anastomotic urethroplasty	2 cm fibrotic occlusion excised; end-to-end anastomosis	Location: Bulbar Stricture Type: Obliterative Length: ~2 cm Etiology: Iatrogenic (cardiac surgery + instrumentation)
2	63	Male	Liver transplant (Nov 2022), failed catheterization, suprapubic cystostomy	T1 & T2: Two strictures in distal bulbous urethra. Post-contrast fat-suppressed T1a sagittal: 2 cm lumen with moderate enhancement (inflammation). Retrograde urethrography/urethroscopy: Complete 2.5 cm bulbous stricture.	Anastomotic urethroplasty	Stricture excised; end-to-end anastomosis	Location: Distal Bulbar Stricture Type: Obliterative Length: ~2.5 cm Etiology: Iatrogenic (liver transplant, catheter-related)
3	58	Male	History of pelvic trauma, multiple endoscopic interventions, suprapubic catheter (2 mo ago)	Gel failed to progress in penile urethra. Distal penile: Two stenotic-occlusive lesions (7 mm, 4 cm & 7 cm from meatus). Proximal penile, bulbar, membranous urethra: patent. Distal prostatic urethra: inadequate expansion over 7 mm.	Anastomotic urethroplasty, Double-face urethroplasty, Dorsolateral Kulkarni	MRI fully concordant with intraoperative findings; three reconstructive techniques employed	Location: Penile + Bulbar (multi-segmental) tricture Type: Multi-segmental, obliterative + non-obliterative Length: 2 × 7 mm obliterations + ~3 cm narrowing Etiology: Traumatic (work accident, dynamite explosion)
4	26	Male	Motorcycle accident (Jun 2024), femur fracture, urinary retention, initial cystostomy → delayed repair (MRI at 5 mo, urethroplasty at 7 mo)	Focal stenotic-occlusive lesion in penile urethra. Located 9 cm from meatus. Proximal urethra preserved.	Anastomotic urethroplasty	Unexpected penile obliteration (posterior typically expected) (<2 cm excised); end-to-end anastomosis with good postoperative flow	Location: Penile Stricture Type: Obliterative Length: <2 cm Etiology: Traumatic (motorcycle accident)

## Data Availability

The data that support the findings of this study are available on request from the corresponding author. The data are not publicly available due to privacy or ethical restrictions.
